# Can mothers rely on the Brazilian health system for their deliveries? An assessment of use of the public system and out-of-pocket expenditure in the 2004 Pelotas Birth Cohort Study, Brazil

**DOI:** 10.1186/1472-6963-8-57

**Published:** 2008-03-18

**Authors:** Aluísio JD Barros, Iná S Santos, Andréa D Bertoldi

**Affiliations:** 1 Programa de Pós-graduação em Epidemiologia, Universidade Federal de Pelotas, RS, Brasil; 2 Programa de Pós-graduação em Saúde Coletiva, Universidade do Vale do Rio dos Sinos, São Leopoldo, RS, Brasil

## Abstract

**Background:**

In a country where comprehensive free health care is provided via a public health system (SUS), an unexpected high frequency of catastrophic out-of-pocket expenditure has been described. We studied how deliveries were financed among mothers of a birth cohort and whether they were an important source of household out-of-pocket expenditure.

**Methods:**

All deliveries occurring in the city of Pelotas, Brazil, during 2004, were recruited for a birth cohort study. All mothers were interviewed just after birth and three months later. Comprehensive data on the pregnancy, delivery, birth conditions and newborn health were collected, along with detailed information on expenses related to the delivery.

**Results:**

The majority of the deliveries (81%) were financed by the public health system, a proportion that increased to more than 95% among the 40% poorest mothers. Less than 1% of these mothers reported some out-of-pocket expenditure. Even among those mothers covered by a private health plan, nearly 50% of births were financed by the SUS. Among the 20% richest, a third of the deliveries were paid by the SUS, 50% by private health plans and 17% by direct payment.

**Conclusion:**

The public health system offered services in quantity and quality enough to attract even beneficiaries of private health plans and spared mothers from the poorest strata of the population of practically any expense.

## Background

Health costs in low and middle income countries can be an important source of expenditure and it has been shown that they can consume a high proportion of family income, up to catastrophic proportions. Brazil was identified as one of the countries with the highest proportion of households suffering from catastrophic health expenditure in a study comparing 59 countries[1]. This study showed that 10% of Brazilian households spent more than 40% of their capacity to pay (as catastrophic expenditure was defined), compared to 6% in Argentina and Colombia, 1.5% in Mexico, 0.5% in the US and practically zero in France and the UK. The only country with a similar result was Vietnam. Such a result is surprising given that Brazil offers comprehensive and free health services to all citizens through its national health system, the SUS (acronym for what could be translated into English as Unified Health System).

The SUS was created in 1988, within the new Constitution, to offer free health care to the population, based on the principles of universal coverage, integral attention and equity. It was a complete change from the previous situation where multiple public systems co-existed for specific groups (urban employees, rural workers, indigents). Since then, the SUS has evolved into a large decentralized system which funds medical care at all levels – including a vast network of primary health care (PHC) units, clinics, emergency services, hospitals and laboratories. Patients also receive a wide range of medicines for free, including full treatment for HIV/AIDS patients. In 2005, the SUS provided more than 600 million consultations and 11.6 million hospital admissions and 152 million doses of vaccine [2]. Service is provided through a mix of public and private institutions – the former typically offering only free public care and the latter both SUS-funded and privately-funded care (for further details on financing see [3]). Maternity care is provided in full by the SUS, including antenatal care (mostly through the PHC network, hospital delivery, neonatal hospital care (ward or intensive care) and post-partum revisions at PHC units. These units also offer pediatric assistance and well-baby clinics.

Despite the fact that SUS provides universal coverage and is expanding their services, the proportion of the Brazilian population covered by private health insurance, or health plan, is around 25%, as estimated by two national surveys done in 1998 [4] and 2003 [5] and by the World Health Survey also carried out in 2003 [6]. In terms of financing health procedures, 60% are paid for by the SUS and the remaining privately, largely through health plans [7]. These are provided by more than 2,000 institutions with national, regional or local coverage. Prices vary considerably, depending on what the service package includes (ambulatory care, hospital care, sophisticated procedures, maternity care) and also on geographic coverage. Private health service providers are regulated by the National Agency of Supplementary Health (ANS) who set the minimum standards of services and a set of rules for contracts. Generally, access to health services through the SUS is high, with less than 5% of those who sought care not succeeding to get it [7]. Almost all deliveries in Brazil take place in a hospital – less than 99% of hospital deliveries are registered only in the North (90%) and Northeast (94%) regions of the country (data from IDB – basic data and indicators, 2004 [8]).

Given the enormous economic and health inequalities observed in Brazil [9], it is important that the SUS is able to offer to the poorest protection against costs that would otherwise be unbearable by the household. The birth of a baby is a critical moment to the family, as the baby is a new source of expenditure, even if healthy. Also, a delivery can be far more expensive than a poor family is able to pay. In Bangladesh [10], the direct and indirect costs of maternity care were studied, along with their affordability by users. Despite deliveries should be virtually free of hospital costs, 51% of the families studied did not have enough money to pay for maternity care, when computing all costs involved (medicines, hospital, travel, etc.). The situation was worse for mothers who had a caesarian section, where 74% were unable to cover expenses. The majority of families in this situation sought help from relatives or friends to borrow money to meet maternity costs.

Health expenditure in Brazil was mostly related to medicines and health plans, according to a study based on a national survey on family budgets [11]. Families among the 20% poorest who had health plans, spent 11–14% of their monthly income with them. Those who had to buy medicines in the study's reference period spent 16–22% of their income with their costs.

Delivery, however, is a relatively rare situation for a given family to be adequately studied in a general survey. We thus used data from a birth cohort initiated in Pelotas, Brazil, in 2004. Data were collected just after the birth and three months later, including information on financing of the delivery and costs incurred by the family. Our objective was to assess which economic groups had expenditure related to the delivery, how much they paid, and, conversely, who was benefiting most from free services provided by the SUS. Coverage by health plans and expenditure with the baby in the three first months of life were also investigated.

## Methods

Pelotas is a medium-sized town (323,000 people, 93% in the urban area according to 2000 National Census, IBGE), located in Southern Brazil. All births from mothers resident in town plus neighboring Jardim América (Municipality of Capão do Leão) during the year of 2004 were recruited for a birth cohort study. Births were identified by daily visits to the five maternity hospitals. Mothers were approached, asked to participate in the study, and after giving written informed consent, they were interviewed using a pre-tested structured questionnaire. Interviews were done in the 24 hours after birth. Information collected about the mother included socioeconomic data, habits, reproductive history, chronic diseases, and antenatal care attendance. Detailed information was collected about the delivery and birth conditions and all newborns were examined. Interviews and exams were done by Nutrition graduates supervised by a pediatrician. The first follow-up visit to the cohort was done within the three days preceding or following the day the child completed three months of age. Enrollment and follow-up rates were very high. More than 99% of deliveries take place at hospital, and the remaining most frequently are taken to hospital in the next few hours for medical assessment. Among the 4,263 mothers approached, only 32 (0.75%) were lost or refused enrolled in the study. In the three-month follow-up 95.7% of the eligible children (i.e. excluding 65 deaths) were interviewed. A detailed account of the study methods and basic description of maternal and child characteristics is given elsewhere[12].

Mother's age, skin color, family income and assets needed to the calculation of a wealth index were collected in the perinatal visit. Skin color was based on self-report to the following categories: white, black, mixed, Asian or Indian. Due to the low frequency of the latter two categories, they were joined in the mixed category. Family income was calculated from adding the values reported by the mothers regarding the income of each person living in the household, plus the values for an additional question on other sources of income (such as government income supplementation programs). The result, in Brazilian Reals (R$), was transformed into minimum wages, worth R$ 260 in July 2004 (approximately USD 90). The IEN (acronym for national economic indicator), an asset-based indicator created through principal components analysis, was calculated from 12 asset variables (such as TV, radio, car) and schooling of the household head [13]. Mothers were classified into reference quintiles, defined by cut-off points derived from the Pelotas population distribution of the indicator. Thus, mothers in the first quintile belonged to the 20% poorest population of the town, and not to the 20% poorest of the sample. The same applies to the other reference quintiles.

Information on delivery financing, coverage by health plan and expenditure was collected at the three-month follow-up. The mothers were asked whether the delivery was paid by SUS, by a health plan or directly to the hospital (out-of-pocket). Mothers were also inquired whether they were covered by a health plan, either private or maintained by a public institution. Any contract to provide health care was considered a health plan, excluding those that simply offer emergency transportation to a health facility. The cost of the plan was recorded as the value effectively paid by the mother or other person living in the household. If someone else paid for the plan, the value recorded was zero, as well as if the plan was offered by the employer as part of a job benefit package. The mother was also inquired about additional payments, such as those related to differentiated accommodation, specialist costs (e.g. anesthesiologists) or under-the-table fees.

Health expenditure with the baby was recorded for the period from birth to the date of the interview. It was inquired separately for medicines, medical consultations, and diagnostic exams. For this analysis all values were summed to give a total expenditure figure, and the proportion for each item calculated for each family.

All declared expenses were reported in Brazilian Reals (R$). The average exchange rate in 2004 was R$ 2.93 per American dollar.

Analyses were done with Stata 9.1 (Stata Corp., College Station, TX, USA, 2006). Associations were tested with χ^2 ^tests, and mean differences between groups were tested by ANOVA or Kruskall-Wallis test, depending on the variable distribution symmetry.

The 2004 Pelotas Birth Cohort waves involved in this paper were approved by the ethics committees from the Federal University of Pelotas Medical School, Brazil, and the WHO, Geneva. All mothers invited to participate were fully informed about the study's aims and procedures. If freely willing to take part, they signed a consent form of which they received a copy.

## Results

In the perinatal study 4189 mothers were interviewed and their description is presented in Table 1. In the three-month follow-up 3946 (94.2%) mothers were interviewed, contributing with information on health plans and health expenditure.

**Table 1:** Description of mothers (N = 4189) recruited to the 2004 Pelotas Birth Cohort Study, Pelotas, Brazil, 2004.

**Variables**	**N**	**%**
Ethnicity (N = 4141)		
white	2555	61.7
black	682	16.5
mixed	904	21.8
Age (years) (N = 4187)		
0–19	796	19.0
20–29	2085	49.8
30–39	1170	27.9
40+	136	3.3
Family income in minimum wages (N = 4177)		
< 1	872	20.9
1 – 2.9	1922	46.0
3 – 6.9	938	22.5
6 – 9.9	241	5.8
10+	204	4.9
Reference wealth quintiles – IEN* (N = 4186)		
1	1006	24.0
2	859	20.5
3	905	21.6
4	745	17.8
5	671	16.0
Coverage by private health plan (N = 3945)		
No	2602	66.0
Yes	1343	34.0

* National Economic Indicator, an asset index based wealth indicator used to classify families according to its distribution in the city of Pelotas.

Nearly 60% of the mothers were white, followed by mixed race (22%) and black (17%). Nearly 50% of mothers were aged 20 to 29 years, and almost 20% were adolescents. Two thirds of the mothers came from families earning less than three minimum wages. Women in the lowest reference quintile of the wealth index comprised 24% of the sample, while those in the highest were 16%, indicating that pregnancy is more frequent among poorer women.

A third of the mothers was covered by a health plan, either paid by the family or included as a job benefit (Table 1). This coverage was strongly dependent on economic level, as shown in Table 2. Health plan coverage rose from 6.3% among the poorest reference quintile to 78.8% among the richest (p < 0.001). Medical consultations and laboratory exams were included in almost all plans, and equally distributed across economic groups. On the other hand, additional usage fees (usually referred to as "moderating factor", a fixed value charged each time a doctor is seen, for example) were more frequent among the lower quintiles. Coverage for hospital treatment was more frequent in the highest quintiles.

**Table 2:** Coverage, characteristics and cost of private health plans paid by study mothers, stratified by socioeconomic position. 2004 Pelotas Birth Cohort Study, Pelotas, Brazil, 2004.

	Reference wealth quintiles (IEN/Pelotas)					All
	1	2	3	4	5	
	N (%)	N (%)	N (%)	N (%)	N (%)	N (%)

Coverage by private health plan (p < 0.001)	58 (6.3)	155 (18.9)	262 (30.6)	370 (52.0)	498 (78.8)	1343 (34.0)
Services included in health plans						
consultations (p = 0.561)	55 (98.2)	145 (96.7)	250 (98.1)	358 (98.9)	483 (98.2)	1291 (98.2)
lab exams (p = 0.058)	52 (92.9)	136 (91.3)	235 (93.3)	341 (94.7)	477 (96.8)	1241 (94.7)
hospital treatment (p < 0.001)	31 (63.3)	65 (47.5)	97 (40.4)	191 (54.9)	366 (74.9)	750 (59.4)
additional usage fee (p = 0.028)	45 (79.0)	107 (72.3)	176 (69.6)	281 (77.8)	338 (68.7)	947 (72.2)

	Mean	Mean	Mean	Mean	Mean	Mean
Monthly per capita cost in R$* (p < 0.001)	7.58	11.14	12.25	19.69	38.33	25.18

Total population	1006	859	905	745	671	4186

* The mean exchange rate in 2004 was R$ 2.93 per American dollar.

The monthly cost of the health plans showed wide variation. Just over 7% of mothers reported their health plans had no direct costs to them, paid by the employer or other person outside the household. Among the others, per capita monthly cost of the plans varied from just R$ 0.33 to R$ 280 (data not shown). Mean per capita cost was R$ 25.18, with. a positive trend with economic position – means varied from R$ 7.58 in the lowest reference quintile to R$ 38.33 in the highest (Table 2).

In relation to health expenditure with the baby in the first three months of life, 73.8% of the mothers reported non-zero expenditure. This proportion varied from 55% among the poorest to 88% among the richest (p < 0.001). Mean expenditure was about eight-fold greater among the richest (p < 0.001). Mothers not covered by a health plan reported expenses with the baby less frequently, and their mean expenditure was less than half the value reported by those covered by a health plan (Table 3). In contrast, out-of-pocket medical expenditure with the delivery was reported by only 4.6% of the mothers (Table 4). Virtually no mother reported expenditure with delivery in the 40% poorest of the population. Among the 20% richest, 17.4% reported some expenditure. Among those who paid for the delivery, there was no important variation in mean values across economic reference quintiles, with an overall mean of R$ 1908 (data not shown).

**Table 3:** Prevalence and means of out-of-pocket expenditure with the baby in the first three months of life among mothers recruited to the 2004 Pelotas Birth Cohort Study, Pelotas, Brazil, 2004.

	Expenditure with baby		Total population
	N (%)	Mean (R$)*	
Reference wealth quintiles (IEN)**	p < 0.001	p < 0.001	
1	551 (54.8)	16.14	1006
2	625 (72.8)	37.14	859
3	693 (76.6)	45.41	905
4	631 (84.7)	83.61	745
5	591 (88.1)	123.07	671
Covered by private health plan	p < 0.001	p < 0.001	
No	1862 (71.6)	39.35	2602
Yes	1230 (91.6)	98.37	1343
All	3093 (73.8)	55.98	4189

* Population average, including households with and without actual spending.

** Classified according to reference quintiles estimated for Pelotas.

**Table 4:** Financing of deliveries and related out-of-pocket (OOP) expenditure among mothers recruited to the 2004 Pelotas Birth Cohort Study, Pelotas, Brazil, 2004.

	Financing mode*			OOP expenses*
	SUS	Health plan	Private	with delivery
	N (%)	N (%)	N (%)	N (%)
Reference wealth quintiles (IEN)**				
1	906 (98.5)	13 (1.4)	1 (0.1)	1 (0.1)
2	782 (95.4)	36 (4.4)	2 (0.2)	2 (0.2)
3	773 (90.5)	64 (7.5)	17 (2.0)	17 (2.0)
4	523 (73.5)	145 (20.4)	44 (6.2)	50 (7.0)
5	213 (33.7)	312 (49.4)	107 (16.9)	110 (17.4)
				
Covered by private health plan				
No	2537 (97.7)	--	60 (2.3)	66 (2.5)
Yes	660 (49.2)	570 (42.5)	112 (8.3)	115 (8.6)
				
Health plan covers hospitalization***				
No	418 (86.4)	--	66 (13.6)	67 (13.8)
Yes	188 (24.2)	544 (69.6)	46 (5.9)	48 (6.2)
				
All	3197 (81.1)	570 (14.5)	172 (4.4)	181 (4.6)

* all cross-tabulation χ^2 ^tests with p < 0.001

** Classified according to reference quintiles estimated for Pelotas.

*** Only mothers who were covered by a private health plan

The Brazilian public health system (SUS) paid for 81.1% of the deliveries, 14.5% were paid by health plans and 4.4% by direct payment (Table 4). In the first and second reference quintiles 98.5 and 95.4% of deliveries were financed by the SUS respectively. In the richest quintile this proportion was down to a third (p < 0.001). Conversely, deliveries paid for by health plans were about a half in the richest quintile and less than 5% among the 40% poorest. Additional payments by SUS patients are not allowed by the system. Only 9 among 3197 mothers (0.28%) reported such payments (data not shown). Nearly 6% of women covered by a health plan including hospital treatment reported paying for the delivery. This might happen because either the hospital of choice or the obstetrician were not included in the insurance coverage.

Among those mothers who were not covered by a health plan, 97.7% had their deliveries through SUS, as expected. Interestingly, nearly half (49.2%) of those mothers who had a health plan were also financed by the SUS (Table 3). This percentage fell to 24.2% among mothers with a health plan covering hospital treatment. Among those with plans not covering hospitalization, 86.4% were financed by the SUS.

Mothers not covered by a health plan reported expenses with delivery less frequently than those who were covered (Table 3). Comparing costs of delivery by type, caesarian sections had a cost marginally higher, of R$ 1952, compared to vaginal delivery, at R$ 1518 (p = 0.045).

## Discussion

The inclusion of all births from mothers living in Pelotas and Jardim América in 2004 and the very low rate of losses (5.8%) are an assurance of data quality in this study. The sample size is large and allows necessary stratifications for the analyses. Additionally, the recall period for health plan and expenditure information was short (three months) and equal for all mothers, contributing to good quality data. It should be noted, however, that information on expenditure and characteristics of health plans were obtained from the mothers, who may not be aware of the exact details of coverage and costs. As in most surveys, information on expenditure was based on recall and thus subject to errors in the reported figures.

The percentage of mothers covered by health plans was similar to estimates for the whole population in Southeastern Brazil [14], and nine percentage points higher than estimates based on national surveys [5,6]. A study based on data from 2003 [5] reported 2.4% and 71% of individuals covered by health plans, among the 20% poorest and 33% richest respectively. This coverage is similar to our finding for the richest, but slightly lower than our result for the poorest. The same study [5] found high proportions of health plans affording for medical consultations and laboratory exams, in agreement with our results. Hospital treatment, however, was covered by health plans in a much higher proportion (92.1% against the 59.4% we found) in this study. This may be due to the considerable number of low-cost health plans offered by independent clinics and worker unions in Pelotas. This is also probably related to the mean costs of health plans reported by the mothers which were much lower than the values reported by Travassos et al [14], ranging from R$ 123 to R$ 361.

The most important results found in this study relate to the high proportion of deliveries financed by the SUS, and the very low proportion of mothers reporting expenses related to delivery. Similar proportions of deliveries financed by the SUS, around 80%, were reported for two national surveys done in 2003 [5,6], suggesting that our results, despite based on data from one municipality may well be valid for the whole country. Even among those mothers covered by health plans, the SUS financed half the deliveries. Also, 25% of the deliveries among mothers with health plans which included hospitalization were financed by the SUS. These proportions are higher than those described for general health care [9]. The reason for this finding may be related to health plans that do not cover specifically maternity care, or simply error in information about health plan coverage by the mother. But it may also be a result of mothers perceiving maternity care offered by the SUS as good quality, or more convenient in terms of hospital location. Unfortunately, mothers were not inquired about such reasons, and further investigation would be needed to satisfactorily answer this question.

In terms of equity promotion, and protection of the poorest from out-of-pocket health expenditure, we have shown here that more than 95% of the 40% poorest mothers had their deliveries financed by the SUS. Also, the number of mothers in this group reporting delivery-related expenses was negligible. The SUS also financed the great majority of deliveries in the third and fourth reference wealth quintiles, reducing its participation to about a third only in the richest quintile. Worth of note is that mothers not covered by a health plan reported delivery-related expenditure less often than those who were covered, indicating that the SUS offered comprehensive coverage for all health needs related to delivery. Additionally, we showed that the poorest mothers also reported less expenditure with their babies in the first three months of life – both in terms of mean value and the proportion of mothers with non-zero expenditure.

Our results make clear that childbirth is not an important cause for catastrophic expenditure. The approach of this study is especially suitable for this assessment since in regular surveys a delivery is a relatively rare event, difficult to be identified in the general picture.

## Conclusion

In summary, we have shown that the majority of deliveries were paid by the SUS, that spared the 40% poorest mothers from expenses. Illegal additional payments within the SUS were practically non-existent. Health plans and direct payment only played an important role in financing deliveries for the 20% richest of the population. Free maternity services offered through the SUS are available to the population, are heavily used and play an important role in reducing health expenditure among the poor and in promoting health equity.

## Competing interests

The author(s) declare that they have no competing interests.

## Authors' contributions

AJB conceived the paper, participated in the design of the study, and drafted the manuscript. IS participated in the design of the study and in drafting the manuscript. ADB carried out the statistical analyses. All authors read and approved the final manuscript.

## Pre-publication history

The pre-publication history for this paper can be accessed here:


